# Expression and Function of IL12/23 Related Cytokine Subunits (p35, p40, and p19) in Giant-Cell Arteritis Lesions: Contribution of p40 to Th1- and Th17-Mediated Inflammatory Pathways

**DOI:** 10.3389/fimmu.2018.00809

**Published:** 2018-04-20

**Authors:** Georgina Espígol-Frigolé, Ester Planas-Rigol, Ester Lozano, Marc Corbera-Bellalta, Nekane Terrades-García, Sergio Prieto-González, Ana García-Martínez, Jose Hernández-Rodríguez, Josep M. Grau, Maria C. Cid

**Affiliations:** ^1^Vasculitis Research Unit, Department of Autoimmune Diseases, Clinical Institute of Medicine and Dermatology, Hospital Clinic, University of Barcelona, Institut d’Investigacions Biomèdiques August Pi i Sunyer (IDIBAPS-CRB CELLEX), Barcelona, Spain; ^2^Vasculitis Research Unit, Department of Emergency Medicine, Hospital Clínic, University of Barcelona, IDIBAPS, Barcelona, Spain; ^3^Department of Internal Medicine, Hospital Clínic, University of Barcelona, IDIBAPS, Barcelona, Spain

**Keywords:** giant-cell arteritis, Th1/Th17 cytokines, IL-12/23 p40, IL-23p19, IL-12p35, glucocorticoid, biologic therapies

## Abstract

**Background:**

Giant-cell arteritis (GCA) is considered a T helper (Th)1- and Th17-mediated disease. Interleukin (IL)-12 is a heterodimeric cytokine (p35/p40) involved in Th1 differentiation. When combining with p19 subunit, p40 compose IL-23, a powerful pro-inflammatory cytokine that maintains Th17 response.

**Objectives:**

The aims of this study were to investigate p40, p35, and p19 subunit expression in GCA lesions and their combinations to conform different cytokines, to assess the effect of glucocorticoid treatment on subunit expression, and to explore functional roles of p40 by culturing temporal artery sections with a neutralizing anti-human IL-12/IL-23p40 antibody.

**Methods and results:**

p40 and p19 mRNA concentrations measured by real-time RT-PCR were significantly higher in temporal arteries from 50 patients compared to 20 controls (4.35 ± 4.06 vs 0.51 ± 0.75; *p* < 0.0001 and 20.32 ± 21.78 vs 4.17 ± 4.43 relative units; *p* < 0.0001, respectively). No differences were found in constitutively expressed p35 mRNA. Contrarily, p40 and p19 mRNAs were decreased in temporal arteries from 16 treated GCA patients vs those from 34 treatment-naïve GCA patients. Accordingly, dexamethasone reduced p40 and p19 expression in cultured arteries. Subunit associations to conform IL-12 and IL-23 were confirmed by proximity-ligation assay in GCA lesions. Immunofluorescence revealed widespread p19 and p35 expression by inflammatory cells, independent from p40. Blocking IL-12/IL-23p40 tended to reduce IFNγ and IL-17 mRNA production by cultured GCA arteries and tended to increase Th17 inducers IL-1β and IL-6.

**Conclusion:**

IL-12 and IL-23 heterodimers are increased in GCA lesions and decrease with glucocorticoid treatment. p19 and p35 subunits are much more abundant than p40, indicating an independent role for these subunits or their potential association with alternative subunits. The modest effect of IL-12/IL-23p40 neutralization may indicate compensation by redundant cytokines or cytokines resulting from alternative combinations.

## Introduction

Giant-cell arteritis (GCA) is a granulomatous vasculitis involving large and medium sized-arteries in aged individuals (1, 2). Glucocorticoids remain the cornerstone of remission-induction in GCA. However, about 40–60% of patients relapse when glucocorticoids (GC) are tapered (3–5). Recently, randomized controlled trials have indicated that inhibiting T-cell activation with abatacept (6) and, particularly, blocking interleukin (IL)-6 receptor with tocilizumab are effective in maintaining glucocorticoid-induced remission (7, 8). However, not all patients respond indicating an unmet need for a better understanding of the hierarchy and contribution of additional pathogenic pathways in GCA.

Giant-cell arteritis has been classically considered a T helper (Th)1-mediated disease based on the presence of granulomas and the expression of interferon (IFN)γ and IFNγ-induced products in the arterial lesions (9–14). However, data generated in recent years indicate that Th17-mediated mechanisms also play a significant role in GCA (14–17).

IL-12 and IL-23 are cytokines mainly produced by dendritic cells and macrophages that regulate the development of Th1 and Th17 responses, respectively. IL-12 is a heterodimeric cytokine composed by two subunits, p35 and p40, with a seminal role in Th1 differentiation and IFNγ production. The IL-12p40 subunit may also interact with p19 to conform IL-23, a crucial cytokine in maintaining and expanding Th17 differentiation (18). The observation that IL-12/23p40-deficient mice were resistant to experimentally induced autoimmune diseases suggested that IL-12 and IL-23 play a major role in triggering or maintaining chronic inflammatory diseases (19). According to these models, IL-23 might have a more prominent pro-inflammatory role since IL-12p35-deficient mice may show exacerbated disease (19–22). Currently, several therapeutic agents targeting IL-12, IL-23, or IL-17 are being tested in clinical trials for a variety of immune-mediated diseases (23).

Preliminary studies have shown that IL-12/23p40, IL-12p35, and IL-23p19 subunits are expressed in GCA lesions (24, 25), but their relative expression, combinations and functions have not been addressed. Interestingly, IL-12/23p40 but not IL-12p35 expression was increased in temporal artery biopsies from patients with relapsing GCA after 1 year of treatment (25), indicating independent functions for both subunits and suggesting a role for IL-23 in persistent disease activity (25).

The aims of this study were to investigate IL-12/23p40, IL-12p35, and IL-23p19 subunit expression in GCA lesions and their combination to conform different cytokines, to investigate the effect of glucocorticoid treatment on subunit expression, to analyze the relationship between subunit expression and glucocorticoid requirements, and to explore IL-12/23p40 function in GCA by exposing temporal artery sections to a neutralizing anti-human IL-12/IL-23p40 antibody.

## Materials and Methods

### Patients

The study group consisted of 50 patients with biopsy-proven GCA diagnosed between 1997 and 2006 at our institution (Hospital Clinic, Barcelona). All patients were prospectively evaluated and treated by the authors (Georgina Espígol-Frigolé, Jose Hernández-Rodríguez, Sergio Prieto-González, and Maria C. Cid) with a predefined glucocorticoid-tapering schedule (4, 26). Patients received an initial prednisone dose of 1 mg/kg per day (up to 60 mg/day) for 1 month. Intravenous methylprednisolone pulse therapy (1 g daily for 3 days) was initially administered to patients with recent (<48 h) visual loss. Prednisone was subsequently tapered at 10 mg/week. When reaching 20 mg/day, this dose was maintained for 1–2 weeks and then reduced to 15 mg/day, which was maintained for 1 month. A further reduction to a maintenance dose of 10 mg/day was attempted. If tolerated, a reduction to 7.5 mg/day was tried after 3–6 months. A reduction to 5 mg/day was attempted approximately 3–6 months later and maintained for 1 year, after which a reduction of 1.25 mg/day was preformed every 6 months. Methotrexate at 15 mg/week was added when patients experienced ≥2 relapses or had developed GC side effects. If relapses occurred, prednisone dose was increased by 10–15 mg/day above the previous effective dose.

Clinical data recorded included disease symptoms at the time of diagnosis, number of relapses, and time to complete prednisone discontinuation with no relapse within the following 6 months. Relapse was defined as reappearance of cranial symptoms, polymyalgia rheumatica, systemic symptoms, or anemia that could not be attributed to other conditions, usually accompanied by a rebound in erythrocyte sedimentation rate (ESR) or C-reactive protein (CRP) (4, 27). Isolated fluctuations on ESR or CRP were not considered relapses.

Clinical data of the patients are displayed in Table S1 in Supplementary Material. Thirty-six patients were treatment-naïve and 14 had received prednisone (1 mg/kg/day) for a median of 7 days (range 2–12) before the performance of temporal artery biopsy. Uninvolved temporal arteries from 20 patients (14 women and 6 men) with a median of 77 years (range 64–91) in whom GCA was considered but not confirmed, served as controls. Final diagnoses of these patients are depicted in Table S2 in Supplementary Material.

This study was carried out in accordance with the recommendations of the Ethics Committee of Hospital Clínic (Barcelona), with written informed consent from all subjects. All subjects gave written informed consent in accordance with the Declaration of Helsinki. The study was approved by the Ethics Committee of Hospital Clínic (Barcelona).

### RNA Isolation and cDNA Synthesis

Temporal artery biopsies were embedded in optimal cutting temperature (OCT, Sakura, The Netherlands), snap-frozen in liquid nitrogen, and stored at −80°C. Total RNA was obtained from tissue with TRIzol Reagent (Invitrogen, Thermo Fisher Scientific, Waltham, MA, USA). RNA (1 µg) was reverse-transcribed to cDNA using the High Capacity cDNA Reverse Transcription kit (Applied Biosystems, Foster City, CA, USA).

### Real-Time Quantitative PCR

cDNA was measured by quantitative real-time PCR using specific Pre-Developed TaqMan gene expression assays from Applied Biosystems using the following probes: Hs01073447_m1 (IL-12p35), Hs01011518_m1 (IL-12p40), Hs00372324_m1 (IL-23p19), Hs00989291_m1 (IFNγ), Hs00171138_m1 (CXCL11), Hs00171042_m1 (CXCL10), Hs00171065_m1 (CXCL9), Hs00174383_m1 (IL-17), Hs00174131 (IL-6), Hs01555410_m1 (IL-1β), and Hs02621508_m1 (tumor necrosis factor, TNFα). All samples were normalized to the expression of the housekeeping gene, GUSb. The comparative CT method was used to assess relative gene expression. Results were expressed as relative units.

### Measurement of Circulating IL-12, IL-12p40, and IL-23p19

Plasma citrate from the 50 GCA patients was obtained at the time of diagnosis before the initiation of glucocorticoid therapy and frozen at −80°C. Plasma from 20 age- and sex-matched healthy donors was obtained for comparison. Human heterodimer IL-12 and IL-12/23p40 subunit were measured in plasma by immunoassay (Quantikine ELISA kits from R&D Systems, Minneapolis, MN, USA). To quantify plasma IL-23, Abcam kit (Cambridge, UK) detecting the IL-23p19 subunit was employed. Procedures were performed according to the manufacturer’s protocol.

### Immunofluorescence Staining and Confocal Microscopy

For qualitative assessment of cytokine distribution at the cellular level, immunofluorescence staining was performed in six temporal artery biopsies obtained from four patients and two controls. Temporal artery fragments were fixed in cold 4% paraformaldehyde (PFA) in phosphate-buffered saline (PBS), cryo-protected in increasing concentrations of saccharose (15 and 30%), embedded in OCT, and frozen at −80°C. Slides with 10-µm cryostat sections were further fixed with 4% paraformaldehyde for 10 min at room temperature, washed in PBS, and soaked in PBS with 0.1% Triton, 1% bovine serum albumin, and 5% donkey serum (Sigma-Aldrich, St. Louis, MO, USA) for 1 h at 4°C. Sections were incubated at 4°C overnight with the following primary antibodies: human IL-12 p35 (mouse monoclonal, Acris-Antibodies, Herford, Germany) at 1:100 dilution, human IL-23p19 (rabbit polyclonal, Abcam) and human IL-12p40 (goat polyclonal, Santa Cruz Biotechnology, Dallas, TX, USA), both at 1:50 dilution, and incubated overnight at 4°C.

Slides were washed in PBS (5 min, three times), followed by incubation with secondary antibodies (spectrally distinct Alexa Fluor-conjugated antibodies to goat, mouse and rabbit IgG; Molecular Probes, Thermo Fisher Sicentific). Nuclei were visualized with Hoechst. Slides were mounted with Prolong Gold Antifade Reagent (Molecular Probes, Thermo Fisher Sicentific) and examined using a laser scanning confocal Leica TCS SP5 microscope (Leica Microsystems, Heidelberg, Germany). Images were processed with Image J software (Wayne Rasband, Bethesda, MD, USA).

### Proximity Ligation Assay (PLA)

Proximity ligation assay was used to visualize close colocalization (<40 nm) of p40 and p19 subunits (IL-23) and p40 and p35 subunits (IL-12), respectively, in temporal artery biopsies using Duolink Detection kit (Olink Bioscience). Tissues were fixed (4% PFA for 20 min at room temperature). After 1-h incubation in blocking buffer (0.1% Triton X-100, 5% FBS 1% BSA in PBS) at room temperature, slides were incubated overnight at 4°C with anti-human p19 rabbit polyclonal Ab (2 µg/ml, Sigma-Aldrich) and anti-human p40 goat polyclonal Ab (10 μg/ml, Santa Cruz Biotechnology) in blocking buffer or alternatively with anti-human p35 rabbit poyclonal Ab (5 µg/ml Atlas, Bromma, Sweden) and anti-human p40 goat polyclonal Ab (10 µg/ml Santa Cruz Biotechnology). After two washes (5 min duration) in wash buffer (0.1 M Tris–HCl pH 7.5, 0.5 M NaCl, 5% Tween-20 in ultrapure water), slides were incubated (30 min at 37°C) with PLA probe solution containing anti-rabbit MINUS and anti-goat PLUS Duolink PLA probes. After washing, circularization and ligation of the oligonucleotides in the probes, an amplification step was performed using polymerase solution (100 min at 37°C). After washing and re-fixation in 4% PFA, the slides were mounted with Duolink II Mounting medium containing DAPI and examined using a laser scanning confocal Leica TCS SP5 microscope (Leica Microsystems, Heidelberg, Germany). Images were processed with Image J software (Wayne Rasband, Bethesda, MD, USA).

### Temporal Artery Culture

Temporal artery sections from 10 GCA patients and 10 controls were embedded in Matrigel to ensure prolonged survival and cultured *ex vivo* as described (10, 11, 28, 29) with or without neutralizing anti-human IL-12p40 mouse monoclonal Ab (10 μg/ml R&D Systems), or dexamethasone (DXM) (0.5 µg/ml, Sigma-Aldrich). Each condition was tested in three replicate wells. Biopsies were frozen in TRIzol reagent for RNA extraction.

### Statistical Analysis

Mann–Whitney test, Spearman correlation, and Kaplan–Meier survival curves analyzed with log-rank test were used for statistical analysis using SPSS software, version PASW 18.0.

## Results

### IL-12/23p40 and IL-23p19 Expression is Increased in Temporal Arteries From Patients With GCA

**Figure 1:**
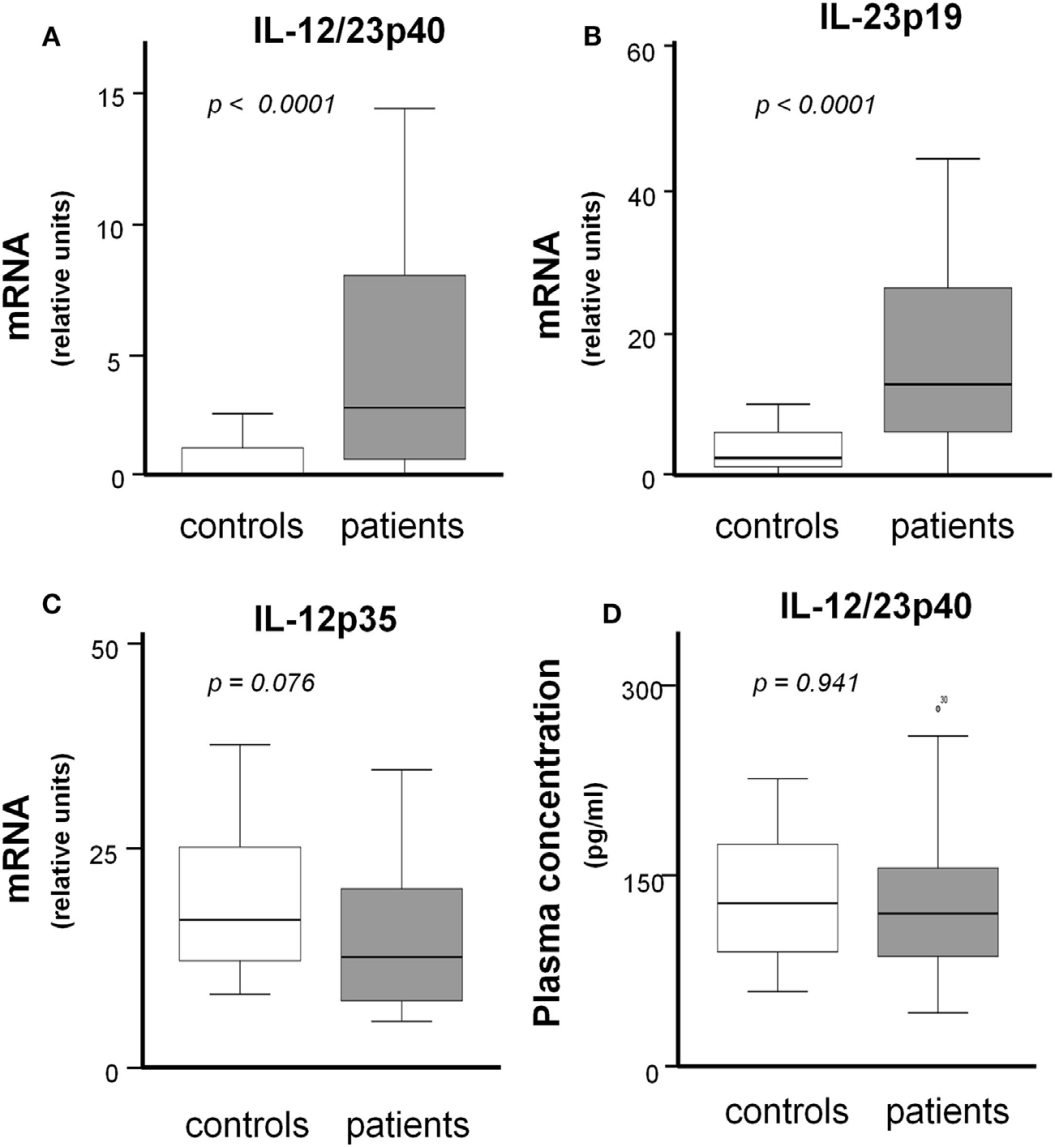
IL-12/23p40, IL-12p35, and IL-23p19 concentrations in temporal artery biopsies (mRNA) and in serum from patients with giant-cell arteritis (GCA). **(A)** IL-12/23p40 mRNA, **(B)** IL-23p19 mRNA, and **(C)** IL-12p35 mRNA expression (relative units) in temporal artery biopsies from 36 treatment-naïve patients and 20 controls. **(D)** IL-12/23p40 concentration in sera from 36 treatment-naïve patients and 19 controls.

As shown in Figures 1A,B, IL-12/23p40 mRNA and IL-23p19 mRNA concentrations were significantly increased in temporal arteries from untreated patients compared to control arteries (4.35 ± 4.06 vs 0.51 ± 0.75 relative units; *p* < 0.0001 and 20.32 ± 21.78 vs 4.17 ± 4.43 relative units; *p* < 0.0001, respectively). By contrast, no significant differences were found in IL-12p35 mRNA expression between patients and controls (14.95 ± 8.9 vs 20.36 ± 14.93, *p* = 0.076) (Figure 1C). IL-12/23p40 mRNA expression in untreated GCA arteries significantly correlated with IL-12p35 (*r* = 0,430, *p* = 0.01) and IL-23p19 (*r* = 0,431, *p* = 0.01).

Plasma IL-12 and IL-23 heterodimeric cytokines were not detectable or were around the detection threshold both in patients and controls (data not shown). Although plasma IL-12/23p40 subunit was measurable, no significant differences between patients and controls were observed (130 ± 95.25 vs 115 ± 50.61 pg/ml; *p* = 0.941) (Figure 1D).

**Figure 2:**
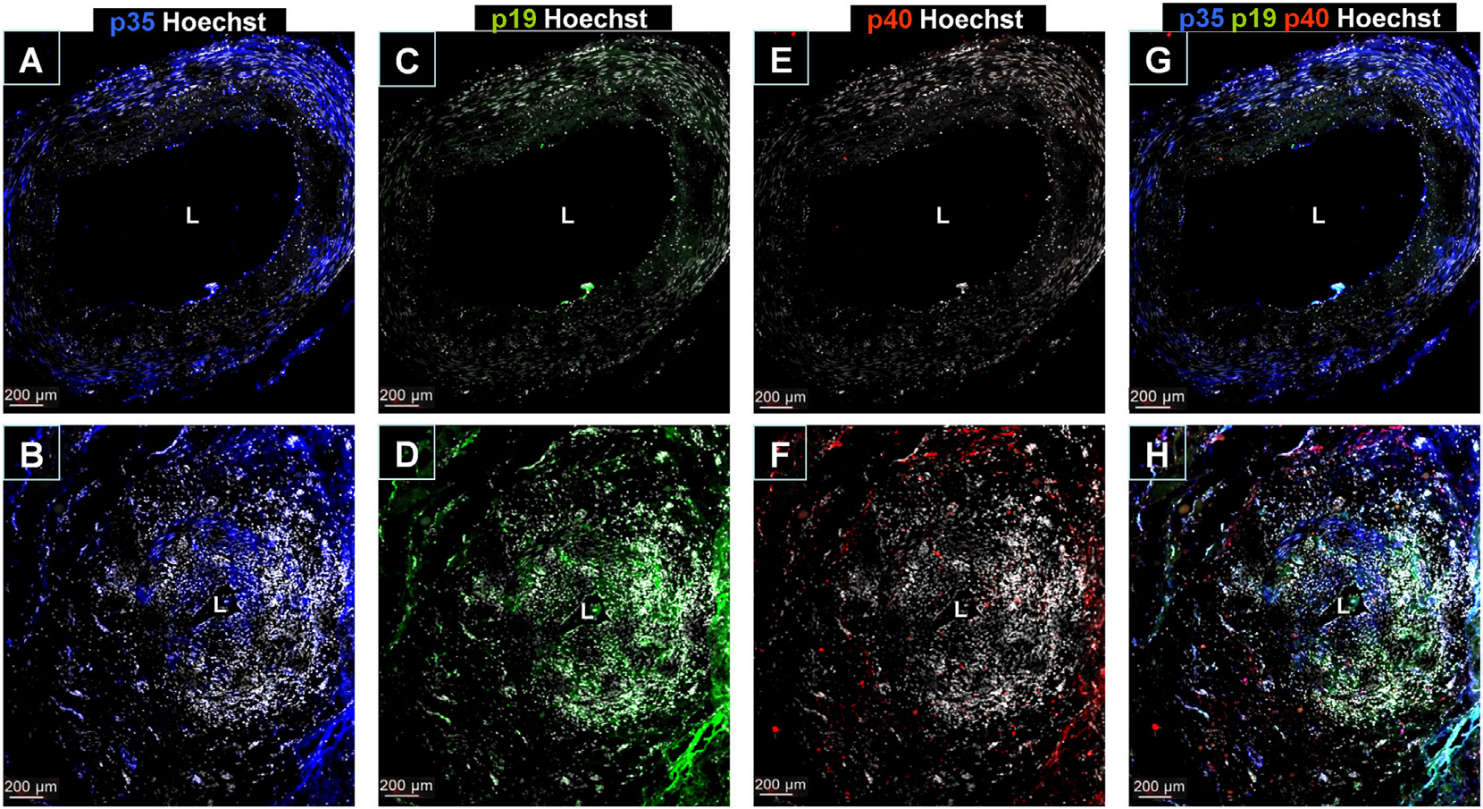
Immunofluorescence detection of IL-12/23p40, IL-12p35, and IL-23p19 subunit expression in temporal artery lesions. **(A)** IL-12p35 staining (blue) in a temporal artery from a control individual showing almost selective expression in the media layer. Nuclei were stained with Hoechst (white). **(B)** IL-12p35 expression in GCA-involved temporal artery section, predominantly in inflammatory infiltrates. **(C,D)** Negative IL-23p19 immunostaining (green) in sections of normal temporal arteries **(C)** and intense expression in GCA samples **(D)** where IL-23p19 expression can be observed in all arterial layers especially in the most inflamed areas. **(E)** Lack of IL-12/23p40 immunostaining in a temporal artery from a control. **(F)** Detection of IL-12/23p40 expression (red) in a GCA-involved temporal artery section predominantly in the adventitial layer. **(G,H)** IL-12p35 (blue), IL-23p19 (green) and IL-12/23p40 (red) staining merge in a temporal artery section from a control and from a GCA patient, respectively. Pictures are representative of four arteries from four GCA patients and two controls, and at least three sections per sample were evaluated.

**Figure 3:**
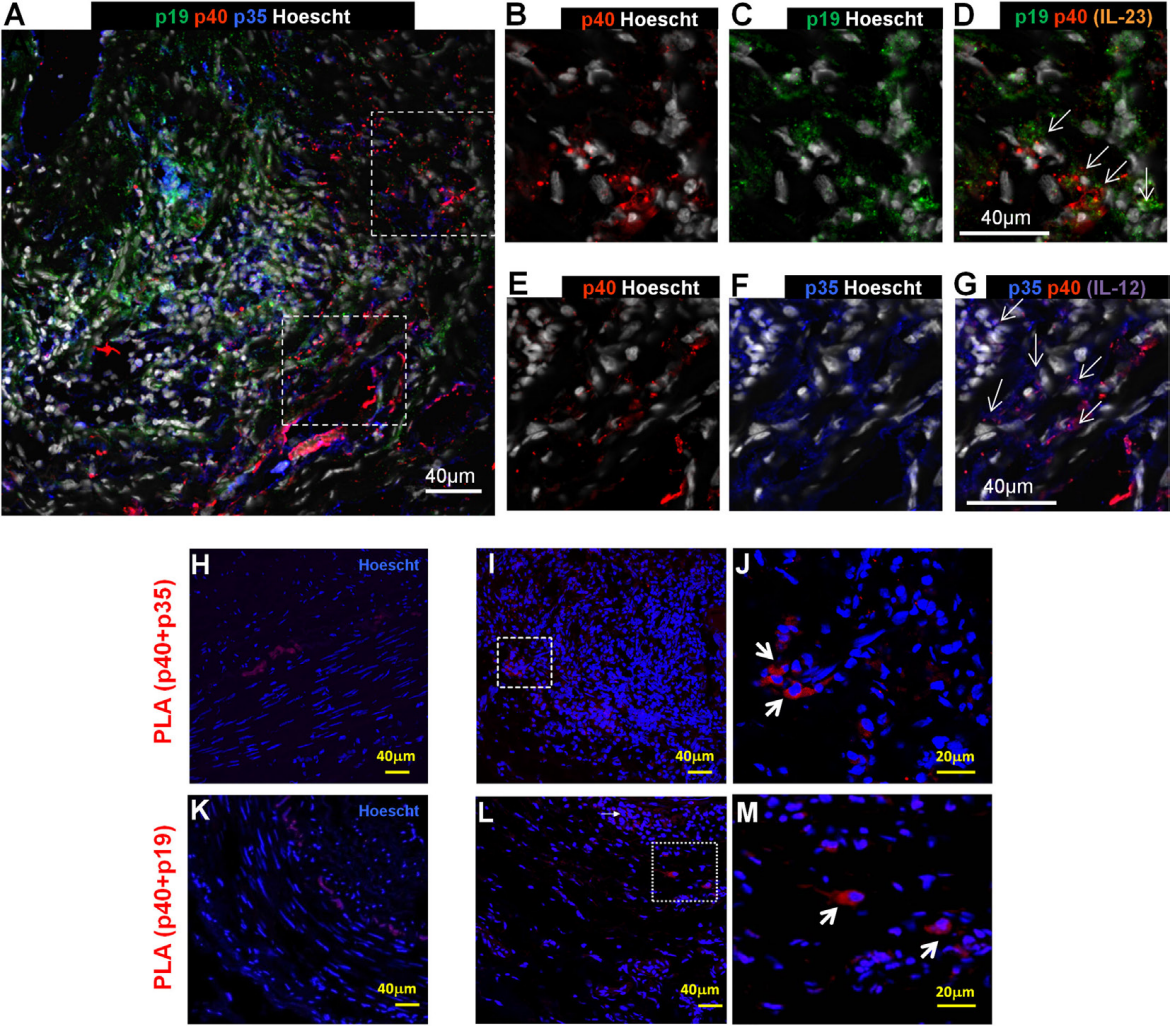
Coexpression of subunits to conform IL-12 and IL-23 in temporal artery lesions from patients with GCA. **(A)** Close-up view of a GCA-involved artery where IL-12p35 + cells (blue) can be observed predominantly in media layer, IL-23p19 + cells (green) in all arterial layers and IL-12/23p40 + cells (red) in the adventitia. **(B)** Higher magnification of inflammatory cells expressing IL-12/23p40 (red), **(C)** IL-23p19 (green), and **(D)** merge showing expression of IL-23 (orange, indicated by arrows). **(E)** Higher magnification of inflammatory cells expressing IL-12/23p40 (red), **(F)** IL12p35 (blue), and **(G)** merge showing expression of IL-12 (purple, indicated by arrows). The temporal arteries were subjected to proximity ligation assay (PLA) to determine proximity of IL-12 and IL-23 subunits. The red staining marks the colocalization (<40 nm) of IL-12/23p40 and IL-12p35 (IL-12). Blue (Hoechst) marks the cell nuclei. **(H)** Negative PLA signalfor colocalization of IL-12/23p40 and IL-12p35 (IL-12) in a control artery. Minimal autofluorescence of the elastic lamina can be appreciated **(I)**. Positive colocalization of IL-12/23p40 and IL-12p35 (IL-12) in a GCA-affected temporal artery. The boxed area is magnified on the right **(J)**. **(K)** Negative PLA signal for colocalization of IL-12/23p40 and IL-23p19 (IL-23) in a control artery. Minimal autofluorescence of the elastic lamina can be appreciated **(L)** Colocalization of IL-12/23p40 and IL-23p19 (IL-23) in a positive GCA temporal artery. The boxed area is magnified on the right **(M)**.

To assess expression of IL-12/23p40, IL-12p35, and IL-23p19 subunits at the protein level and their distribution in temporal arteries, immunofluorescence staining was performed. IL-12p35 had constitutive expression in the muscular layer from normal arteries in accordance with the remarkable IL-12p35 mRNA concentration found in control arteries (Figure 2A). However, IL-12p35 distribution changed in GCA-involved arteries: as the medial layer was damaged, muscular IL-12p35 decreased and it was mainly expressed by infiltrating leukocytes (Figure 2B). IL-12p40 and IL-23p19 protein expression was virtually undetected in normal arteries (Figures 2C,E) and clearly increased in GCA-affected arteries, mostly at the expenses of inflammatory cells (Figures 2D,F). Interestingly, intense p19 or p35 expression by inflammatory cells, independent from p40, could be observed and p19 and p35 were more abundant than p40 (Figure 2). Although p35 and p19 expression exceeded that of p40, association between subunits p40 and p35 or p40 and p19 to conform IL-12 and IL-23, respectively, could be confirmed by PLA in GCA lesions and barely in control arteries (Figure 3).

### IL-12/23p40 and IL-23p19 Expression is Decreased in Temporal Arteries From Glucocorticoid-Treated GCA Patients

**Figure 4:**
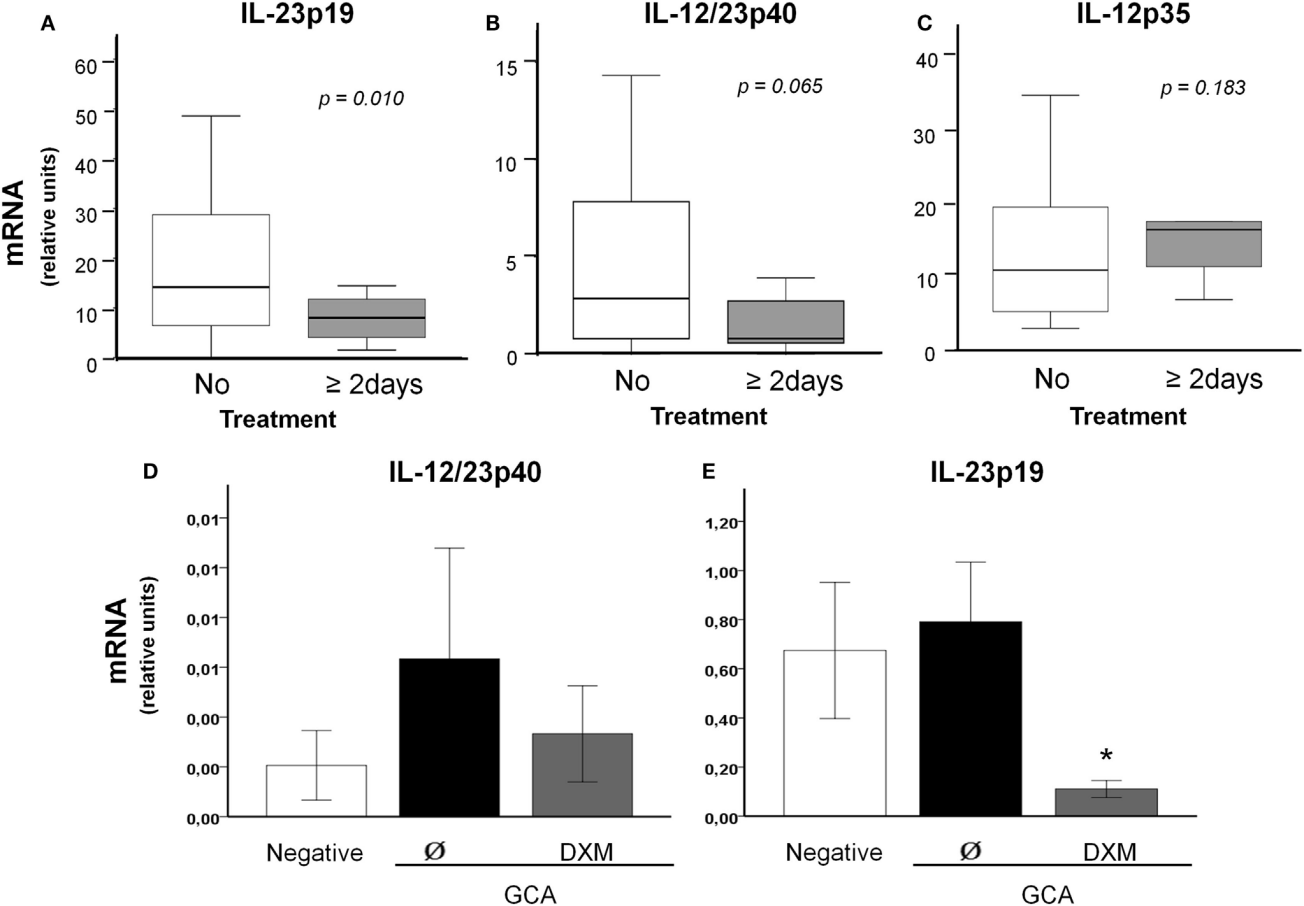
Decreased IL-12/23p40 and IL-23p19 expression in temporal arteries from treated GCA patients. IL-23p19 **(A)**, IL-12/23p40 **(B)**, and IL-12p35 **(C)** expression (relative units) in temporal arteries from 36 treatment-naïve and 14 prednisone-treated GCA patients. **(D)** mRNA IL-12/23p40 expression and **(E)** mRNA IL-23p19 expression on cultured biopsies *(negative and GCA)*, showing the effect of dexamethasone (DXM) in GCA arteries. Bars represent mean ± SEM.

IL-23p19 mRNA concentrations in temporal artery biopsies from treated GCA patients were significantly lower than those found in treatment-naïve GCA patients (7.87 ± 8.04 vs 20.32 ± 21.78 relative units; *p* = 0.010) (Figure 4A). IL-12/23p40 mRNA concentrations also tended to be reduced in treated patients (2.30 ± 3.39 vs 4.35 ± 4.06 relative units; *p* = 0.065) but differences did not reach statistical significance (Figure 4B). No differences were found in IL-12p35 mRNA between both groups (16.57 ± 8.9 vs 14.95 ± 8.9 relative units; *p* = 0.183) (Figure 4C).

The effect of glucocorticoids in reducing p40 and p19 expression was confirmed in cultured temporal arteries from patients with GCA. As with freshly stained arteries, IL-12/IL-23 p40 and IL-23p19 subunits tended to be more abundant in cultured arteries from patients with GCA than in those obtained from controls, although differences did not reach statistical significance, probably due to the small sample size and remarkable individual variability. DXM significantly reduced p19 mRNA and tended to decrease p40 expression (Figures 4D,E).

### Lack of Correlation Between IL12/23p40, IL12p35, and IL23p19 mRNA Expression and GCA Clinical Findings

As shown in Table S3 in Supplementary Material, no differences in IL-12/23p40 or IL-12p35 mRNA expression according to clinical findings could be observed. A trend toward higher expression of IL23p19 mRNA was observed in patients with systemic symptoms (fever or weight loss) (41.46 ± 12.7 vs 17.79 ± 3.7 relative units, *p* = 0.055) but the difference did not reach statistical significance.

### IL-12/23p40, IL-12p35, and IL-23p19 Expression and Long-Term Response to Glucocorticoid Treatment

**Figure 5:**
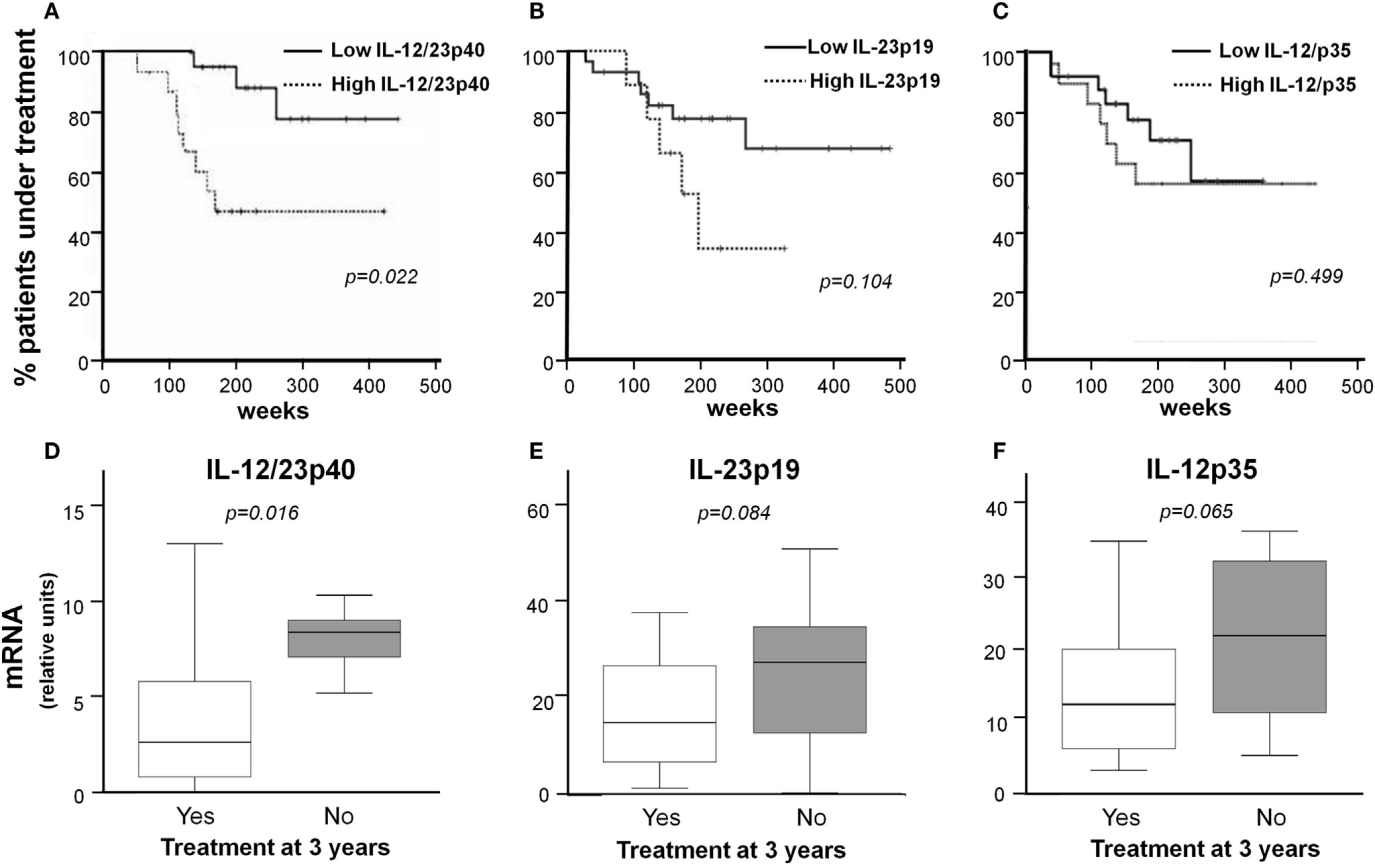
IL-12/23p40, IL-12/23p19, and IL-12p35 mRNA expression in lesions and long-term response to therapy. Percentage of treatment-naïve GCA patients requiring prednisone treatment over time, according to the intensity of IL-12/23p40 **(A)**, IL-12/23p19 **(B)**, and IL-12p35 **(C)** mRNA expression, in temporal artery lesions. High refers to mRNA levels above the 75th percentile (*N* = 26), and low below the 75th percentile (*N* = 10). IL-12/23p40 **(D)**, IL-23p19 **(E)**, and IL-12p35 **(F)** mRNA concentrations in initial temporal artery biopsies from patients still requiring prednisone (*N* = 12) compared with patients in sustained remission (*N* = 24), 3 years after diagnosis.

Patients with elevated IL-12/23p40 mRNA content (above 75% percentile) in their artery lesions were able to completely withdraw prednisone earlier than patients with lower IL-12/IL-23p40mRNA levels (*p* = 0.022) (Figure 5A). Similarly, patients with high IL-23p19 mRNA tended to tolerate prednisone discontinuation earlier than those with lower IL-23p19 RNA values (*p* = 0.104) (Figure 5B). No differences were found between patients with high or low levels of IL-12p35 in terms of glucocorticoid treatment duration (Figure 5C). Accordingly, IL-12/IL-23p40 mRNA levels were significantly higher in patients able to completely discontinue prednisone at 3 years (7.37 ± 2.25 vs 4.29 ± 4.58 relative units; *p* = 0.016) (Figure 5D). No significant differences were found in IL-12p35 and IL-23p19 mRNA concentrations between patients requiring or not prednisone at 3 years (20.8 ± 10.54 vs 14.07 ± 8.74 relative units; *p* = 0.065 and 29.59 ± 25.72 vs 15.03 ± 11.25 relative units; *p* = 0.084, respectively) (Figures 5E,F). However, there were no significant differences in IL-12/23p40, IL-12p35, and IL-23p19 mRNA levels in temporal arteries from patients who achieved sustained remission compared with those who presented relapses (4.42 ± 4.3 vs 5.04 ± 4.21 relative units; *p* = 0.516, 15.25 ± 9.46 vs 16.63 ± 9.93 relative units; *p* = 0.682, 20.88 ± 21.59 vs 21.79 ± 22.32 relative units; *p* = 0.616, respectively) (Table S4 in Supplementary Material).

### Effect of Neutralizing IL-12/23p40 Subunit in Cultured Temporal Arteries From Patients With GCA

**Figure 6:**
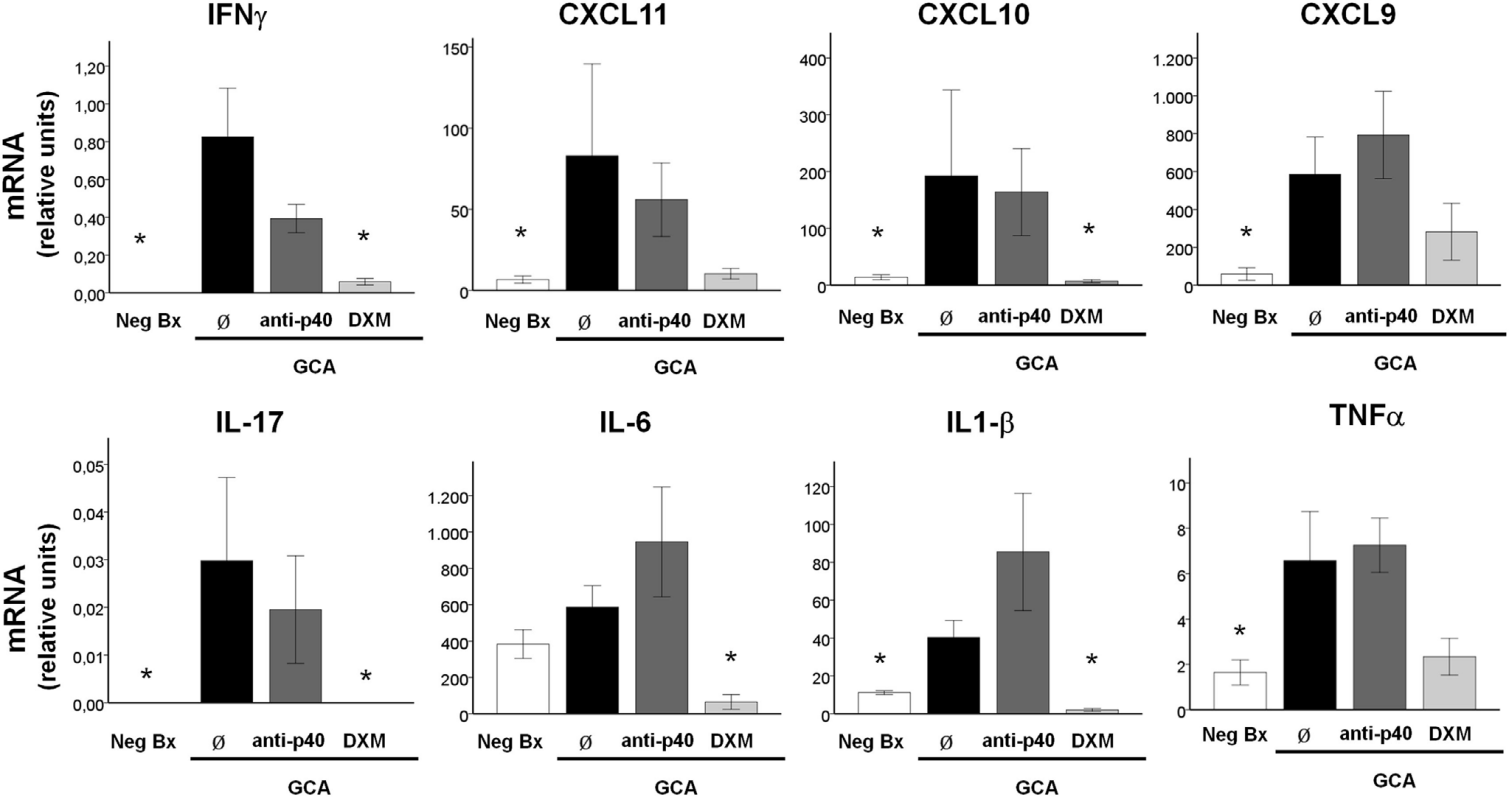
Changes in gene expression by blocking IL-12/23p40 on cultured GCA biopsies. mRNA concentrations of IFNγ, CXCL11, CXCL10, CXCL9, IL-17, IL-6, IL-1β, and tumor necrosis factor α in 10 cultured control arteries (negative biopsies, Neg Bx) vs 10 cultured GCA-involved arteries untreated or exposed to anti-human IL-12p40 mouse monoclonal Ab (anti-p40) (10 µg/ml), or dexamethasone (DXM) (0.5 µg/ml). Statistical comparisons were performed between histologically negative and GCA-involved arteries and between GCA-involved arteries and anti-p40 treated and DXM treated. Bars represent mean ± SEM, *p < 0.05.

In order to elucidate potential functions of the IL-12/23p40 subunit in GCA, we investigated the effects of blocking IL-12/23p40 with a neutralizing monoclonal antibody in *ex vivo* cultured temporal artery biopsies. Several molecules related to Th1 and Th17 differentiation were investigated (9, 11, 30). Neutralization of IL-12/IL-23p40 tended to decrease IFNγ mRNA and, slightly, IFNγ-induced chemokine CXCL11 and CXCL10, but not CXCL9, mRNAs in cultured arteries. IL17 mRNA also tended to decrease and no apparent effect on TNFα expression was observed. Conversely IL-6 and IL-1β mRNA involved in Th17 differentiation tended to increase, possibly as a compensatory mechanism (Figure 6). As shown in the same figure, DXM significantly reduced IFNγ, CXCL10, IL-17, IL-6, and IL-1β and tended to reduce CXCL9 and TNFα.

## Discussion

IL-12/23p40 and IL-23p19 expression was significantly increased in GCA lesions, confirming preliminary observations obtained from small series of patients (24, 25). By contrast, IL-12p35 mRNA expression was similar between patients and controls. However, in normal arteries, IL-12p35 was primarily expressed by vascular smooth muscle cells (VSMC) whereas in GCA arteries, where substantial damage and loss of VSMC frequently occur, IL-12p35 was mostly expressed by inflammatory cells. Similar changes in distribution between normal and inflamed arteries have been observed in other molecules expressed by both VSMC and infiltrating leukocytes (29, 31). Expression of IL-12p35, IL-12/23p40, and IL-23p19 subunits suggest that IL-12 and IL-23 heterodimers are present in GCA lesions. By PLA we could demonstrate, indeed, colocalization of the IL-12/IL-23p40 and IL-12p35 subunits configuring IL-12 and colocalization of IL-12/IL-23p40 and IL-23p19 subunits conforming IL-23 in GCA, supporting the participation of both Th1 and Th17 differentiation pathways in the development of arterial inflammation. However, IL-12/23p40 expression was low, compared to its partners IL-12/p35 or IL-23p19 which were remarkably more abundant. This may explain why initial attempts using less sensitive methods concluded that there was no IL-12/23p40 expression in GCA (32). Distribution of lesions was also dissociated: while IL-23p19 and IL-12p35 subunits were detected in all arterial layers, IL-12/23p40 was mainly found in the adventitia. Consequently, our results indicate that, in GCA, p19 and p35 can be expressed independently from p40 being also part of heterodimeric cytokines other than IL-23 or IL-12, that could be also present in GCA lesions. Accordingly, increased expression of IL-23p19 exceeding the relatively low levels of bioactive IL-23 has also been detected in the rheumatoid synovium (33).

The IL-12/IL-6 family of cytokines resulting from different combinations of alpha (p28, p35, p19) and beta [p40, Ebstein-Barr induced 3 (EBi3)] subunits is currently expanding (34). Recently, a novel IL-12 family pro-inflammatory member named IL-39 composed by IL23p19 and EBi3 and secreted by lipopolysaccharide-stimulated B lymphocytes has been described (34, 35). Moreover IL-12p35 may combine with EBi3 to conform IL-35, a putatively suppressive cytokine produced by regulatory T lymphocytes (34, 36). Adding complexity, some subunits may be individually functional without interaction with partner subunits: we have recently demonstrated that IL-23p19, but not IL-12/23p40, can be expressed by endothelial cells exposed to inflammatory stimuli. Endothelial p19 has a p40-independent role as an intracellular activator of endothelial cells, by directly interacting with endothelial gp130 and leading to phosphorylation of signal transducer and activator of transcription3 (37). As observed in GCA, IL-23p19 mRNA is more abundant than IL-12/IL-23p40 mRNA in cultured macrophages (18), raising the possibility that p19 may play independent functional roles even in cells such as macrophages able to secrete mature, heterodimeric, IL-23.

Although both IL-12/and IL-23 were increased in lesions, mature IL-12 and IL-23 were barely detectable in plasma suggesting that functional activities of these cytokines are predominantly local. IL-12/23p40 subunit, which is known to form homodimers with a presumed counter-regulatory role (34, 38) was present in plasma but with no significant differences between patients and controls. Contrarily to other cytokines such as TNFα or IL-6 which have systemic effects (30), and consistent with the local paracrine effects of IL-12 and IL-23 subunits, there was no significant relationship between their tissue expression and disease manifestations.

IL-12/23p40 and, particularly, IL-23p19 were reduced in biopsies from treated patients in accordance with our previous results in a small series (25). Moreover, downregulation by glucocorticoids was confirmed in cultured arteries. Interestingly, patients with strong initial expression of IL-12/23p40 were able to discontinue prednisone treatment earlier than patients with low IL-12/23p40 expression and IL-12/23p19 followed a similar trend. Given that IL-23 is essential for the expansion and homeostasis of Th17 cells (39, 40), this finding is consistent with a previous study where we found that a strong IL-17A expression was associated with more sustained response to glucocorticoids, suggesting that patients who develop a predominantly Th17 response are more sensitive to glucocorticoid treatment (17). Downregulation of these pro-inflammatory cytokines may partially account for the therapeutic relief provided by glucocorticoids.

Although not highly expressed in lesions, IL-12/23 p40 may be relevant to vascular inflammation in large-vessel vasculitis. Recently, a meta-analysis of massive genotyping studies performed with GCA and Takayasu arteritis patients showed that a variant in close proximity to the *IL-12B* gene (encoding for IL12/23p40) is associated with increased genetic risk for both diseases, although the putative functional impact of this variant on *IL-12B* expression remains unknown (41). A recent open-label trial with ustekinumab, a monoclonal antibody neutralizing IL-12/23p40, suggests benefit in a small series of patients with refractory/relapsing GCA (42). Analysis of the peripheral blood compartment revealed reduction in both Th1 and Th17 polarization in a patient with GCA upon ustekinumab treatment (43). However, when we analyzed the effects of blocking IL-12/23 p40 on involved tissue from GCA patients, only a trend, consistent with its known biology, was observed. IL-12/23p40 inhibition tended to reduce IFNγ expression as well as expression of IFNγ induced chemokines CXCL10 and 11 but not CXCL9. IL-12/23p40 blockade slightly reduced IL-17 expression although, interestingly, cytokines involved in Th17 differentiation such as IL-1β and IL-6 increased, possibly as a compensatory mechanism. Effects in tissue may be more complex than those observed in the peripheral compartment (43) and stimuli and interactions with other elements in the microenvironment may configure a protective niche. Although the inhibitory effect of the anti-IL-12/23p40 antibody used in our study may not be equivalent to that of ustekinumab, which has been generated for therapeutic purposes, recent studies suggest that neutralizing IL-12/23p19 may have more potent effects than blocking IL-12/23p40 in suppressing inflammatory activity in other diseases (44).

Our functional *ex vivo* model has some limitations such as isolation from a functional immune system or induction of changes by the culture itself in the expression of some inflammatory molecules (10). Moreover, lesions are often segmental in GCA arteries and this may have increased variability in responses. However, in spite of these limitations this model has been useful to evidence functional modifications after therapeutic intervention with various agents, including biologic agents (10, 11).

The increasingly recognized diversity of subunits configuring the IL-12/IL-6 superfamily of cytokines to which IL-12 and IL-23 belong, as well as the multiple potential partnership of subunits and receptor chains providing pro-inflammatory and anti-inflammatory stimuli depending on the specific combinations, has added an unexpected complexity to this system (34). Our results clearly indicate that additional combinations to the classical heterodimers IL-12 and IL-23 may occur in GCA and may lead to incomplete or compensated responses to the blockade of single subunits.

## Ethics Statement

The study was approved by the Ethics Committee of Hospital Clínic (Barcelona). All subjects gave written informed consent in accordance with the Declaration of Helsinki.

## Author Contributions

GE-F, EP-R, and MC conceived experiments and interpreted data, GE-F, EP-R, and EL carried out experiments. GE-F and AG-M collected patient data. GE-F, JH-R, SP-G, and MC provided patient care. GE-F and EP-R generated figures. GE-F and MC wrote the manuscript. All authors were involved in final approval of the submitted version.

## Conflict of Interest Statement

MC has received consulting fee from Roche. All other authors declare that the research was conducted in the absence of any commercial or financial relationships that could be construed as a potential conflict of interest.
