# Supplementary material for: Expression and Function of IL12/23 Related Cytokine Subunits (p35, p40, and p19) in Giant-Cell Arteritis Lesions: Contribution of p40 to Th1- and Th17-Mediated Inflammatory Pathways
Source: Front Immunol. 2018 Apr 20;9:809. doi: 10.3389/fimmu.2018.00809 (PMC5920281; doi:10.3389/fimmu.2018.00809)
Supplement: Supplementary file 1 [file table_1.PDF]

Table S1. Clinical features of giant cell arteritis (GCA) patients

	N
**General features**	
Age median	78 (58-91)
Sex (female/male)	45/12
**Cranial symptoms**	**43(86%)**
headache	36
jaw claudication	10
hypersensitivity scalp	9
facial pain	3
ocular pain	3
tongue pain	4
ear pain	3
carotidynia	2
toothache	2
odynophagia	9
Abnormal temporal artery*	33
**Ischemic events**	**12(24%)**
permanent lost of vision	6
amaurosis fugax	4
transient diplopia	1
stroke	1
**Systemic manifestations**	**31(62%)**
fever	22
weight lost	28
**Polymyalgia rheumatica**	**21 (42%)**

*Abnormal temporal artery: painful, swollen, with decreased or absent pulse.
