# Supplementary material for: Expression and Function of IL12/23 Related Cytokine Subunits (p35, p40, and p19) in Giant-Cell Arteritis Lesions: Contribution of p40 to Th1- and Th17-Mediated Inflammatory Pathways
Source: Front Immunol. 2018 Apr 20;9:809. doi: 10.3389/fimmu.2018.00809 (PMC5920281; doi:10.3389/fimmu.2018.00809)
Supplement: Supplementary file 2 [file table_2.PDF]

Diagnosis of the control patients with negative temporal artery biopsy

The 20 patients with negative temporal artery biopsy (TAB) were consecutively selected among negative temporal artery biopsies when the suspicion of giant cell arteritis (GCA) by the treating physicians was considered low. Low suspicion was defined as the absence of intention to treat as GCA unless a positive biopsy was obtained. Temporal artery biopsy was performed in these patients to reasonably rule out rather to confirm GCA.

Table S2. Demographic data and final diagnosis in control patients

Demography (20 patients)

Age,years	77 (64-91)
Gender (male/female) ratio	6/14
**Diagnosis**	
Non-arteritic anterior ischemic neuritis	1
Chronic multifactorial anemia	2
Non-specific headache in pluripathologic patients	8
Non-specific headache with elevation of acute-phase reactants that were self-limited	3
Infection	2
Myelodysplastic Syndrome	1
Small Vessel vasculitis	1
Polymyalgia Rheumatica (no GCA symptoms after 2 years follow-up)	1

Except for the 2 patients with Polymyalgia rheumatica, and the patient with systemic small vessel vasculitis none of the controls received long-term glucocorticosteroid treatment.
