# Supplementary material for: Expression and Function of IL12/23 Related Cytokine Subunits (p35, p40, and p19) in Giant-Cell Arteritis Lesions: Contribution of p40 to Th1- and Th17-Mediated Inflammatory Pathways
Source: Front Immunol. 2018 Apr 20;9:809. doi: 10.3389/fimmu.2018.00809 (PMC5920281; doi:10.3389/fimmu.2018.00809)
Supplement: Supplementary file 3 [file table_3.PDF]

Table S3. IL-12p40, IL-12p35 and IL-23p19 expression in temporal artery lesions from patients with giant-cell arteritis according to clinical findings

mRNA Relative units	**Cranial Symptoms**			**Polymyalgia rheumatica**			**Systemic Symptoms**			**Cranial ischaemic complications**		
	Present	Absent	*p*	Present	Absent	*p*	Present	Absent	*p*	Present	Absent	*p*
**IL-12/Il23p40**	3.98±1.75	4.96±0.30	***0.057***	4.25±0.84	4.67±1.3	*0.68*	4.16±1.56	4.72±0.75	*0.64*	3.20±1.05	4.89±0.89	*0.14*
**IL-12p35**	14.24±4.75	17.23±2.20	*0.15*	21.65±0.95	16.17±1.22	*0.35*	22.03±5.07	15.46±1.93	0.24	11.21±1.83	19.38±2.73	*0.07*
**IL-23p19**	22.39±4.62	18.89± 3.34	*0.23*	35.74±10.3	19.27±4.22	*0.43*	41.46±12.7	17.79±3.7	**0.055**	16.21±7.01	29.18±6.47	*0.60*
