# Supplementary material for: Expression and Function of IL12/23 Related Cytokine Subunits (p35, p40, and p19) in Giant-Cell Arteritis Lesions: Contribution of p40 to Th1- and Th17-Mediated Inflammatory Pathways
Source: Front Immunol. 2018 Apr 20;9:809. doi: 10.3389/fimmu.2018.00809 (PMC5920281; doi:10.3389/fimmu.2018.00809)
Supplement: Supplementary file 4 [file table_4.PDF]

Table S4. IL-12p40, IL12-p35 and IL23-p19 expression in temporal artery lesions from patients with giant-cell arteritis according to relapses

	Relapses		
	0	≥ 1	
**IL-12/23p40 mRNA**	4.41 ± 4.3	5.04 ± 4.21	*p 0.516*
**IL-12p35 mRNA**	15.25 ± 9.46	16.63 ± 9.93	*p 0.682*
**IL-23p19 mRNA**	20.88 ± 21.59	21.79 ± 22.32	*p 0.641*
